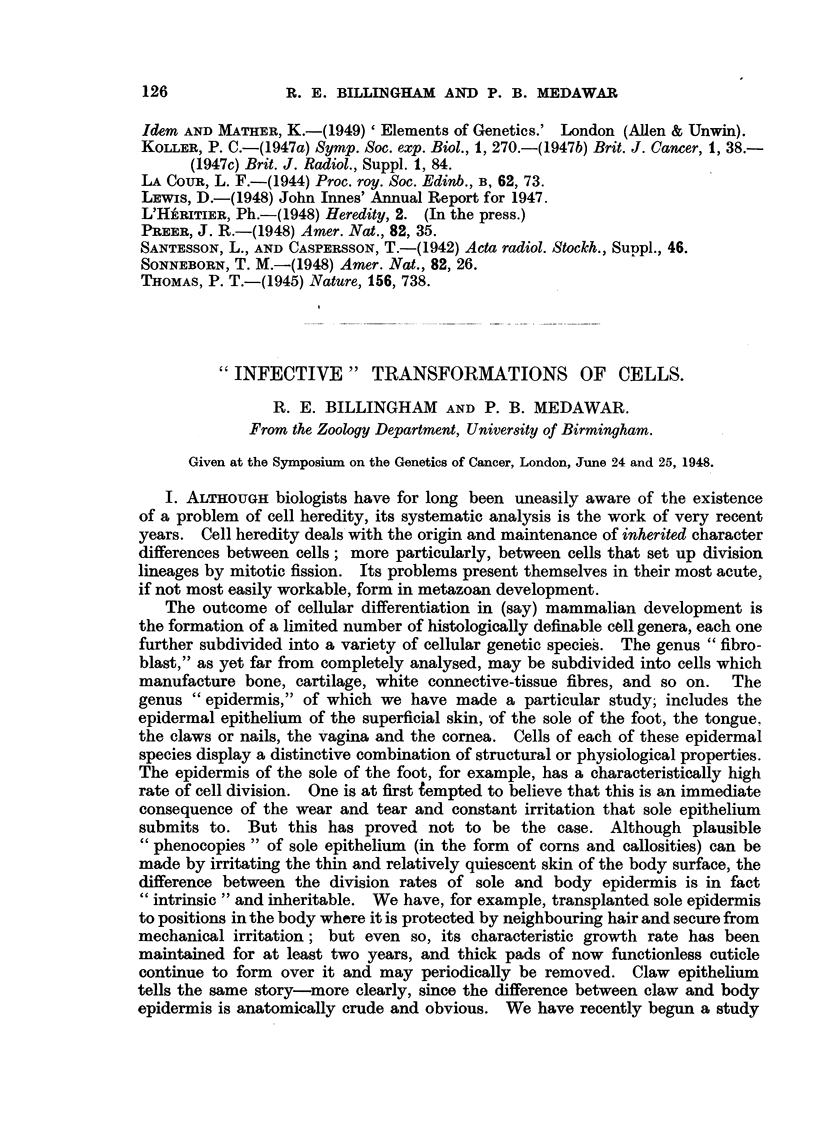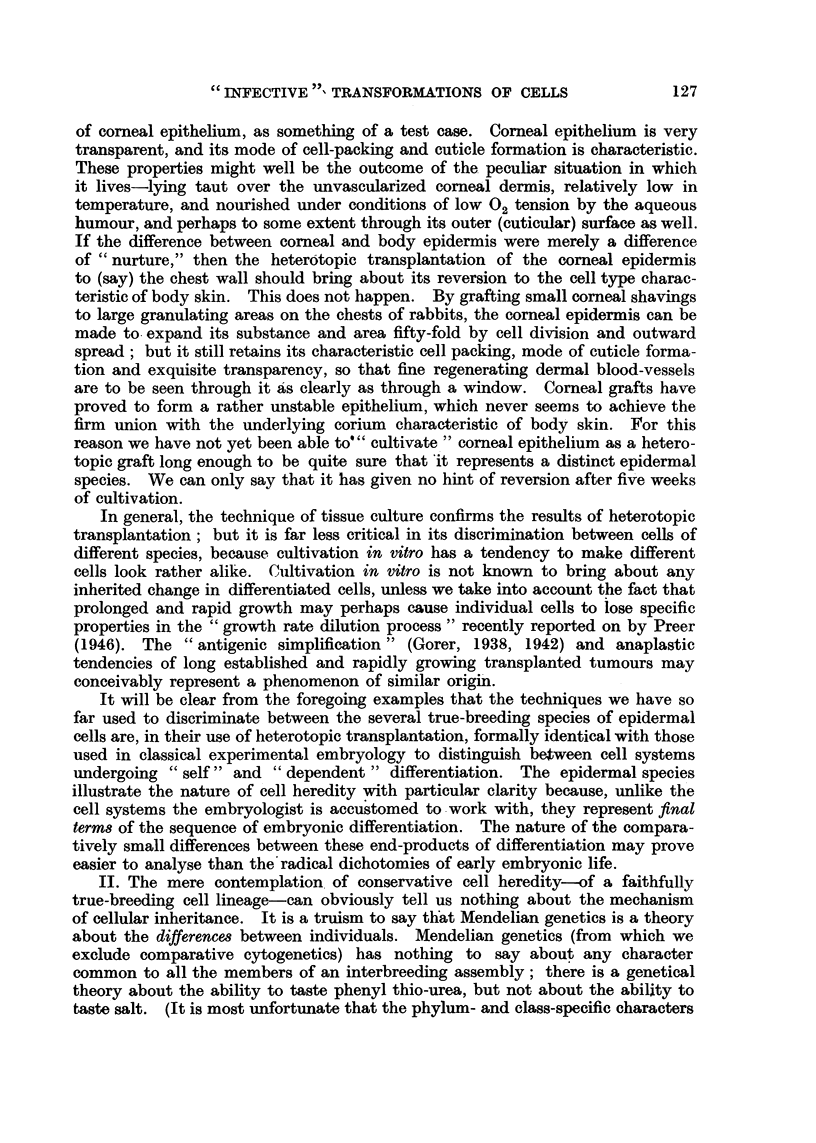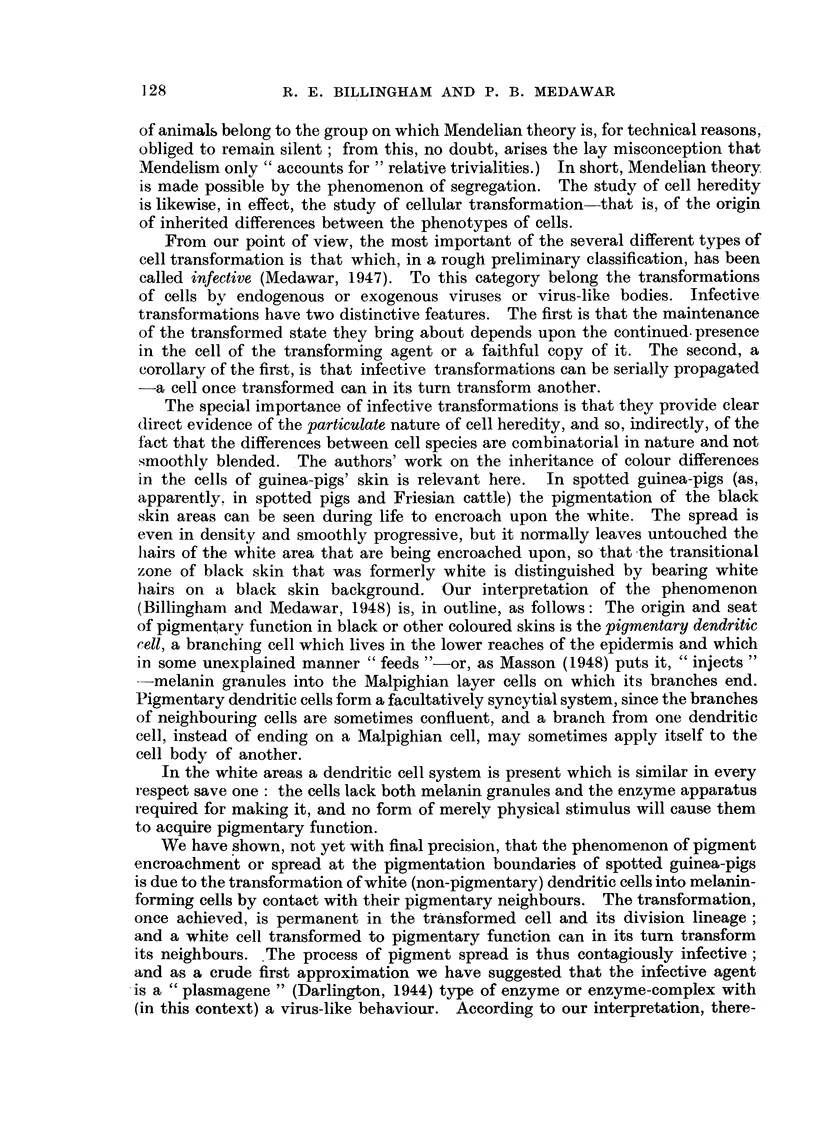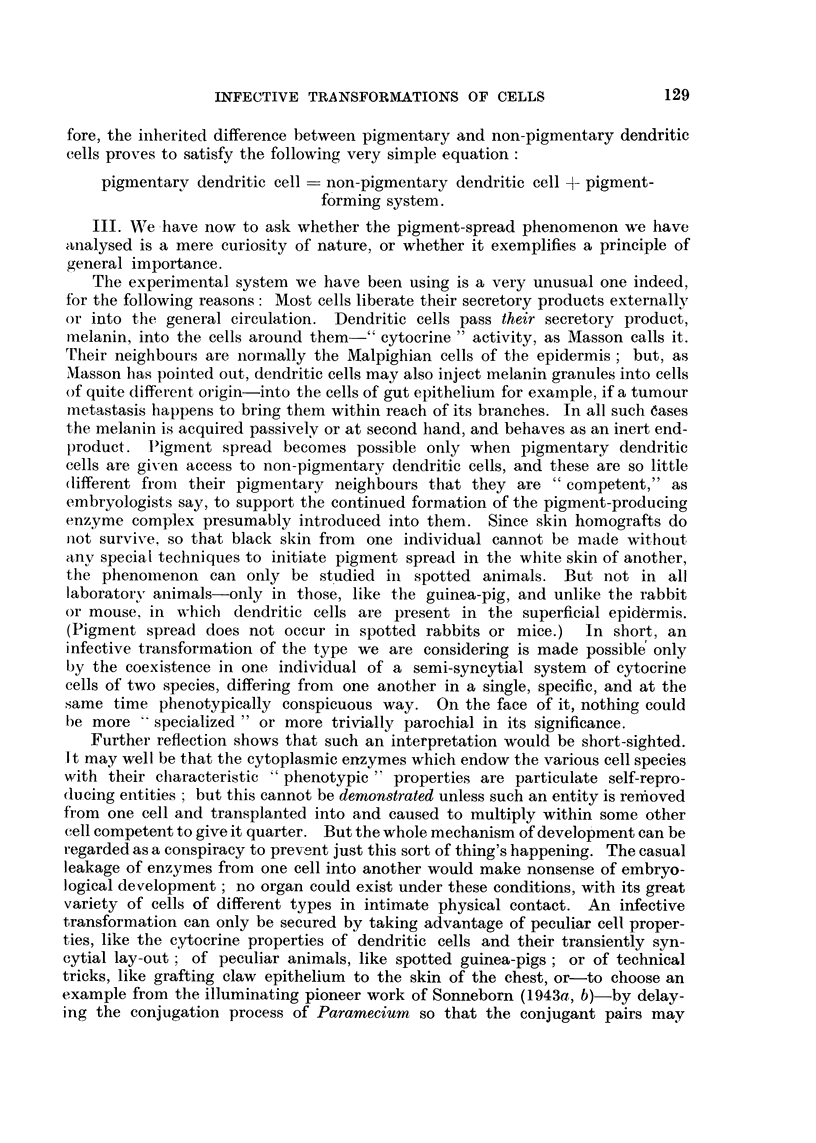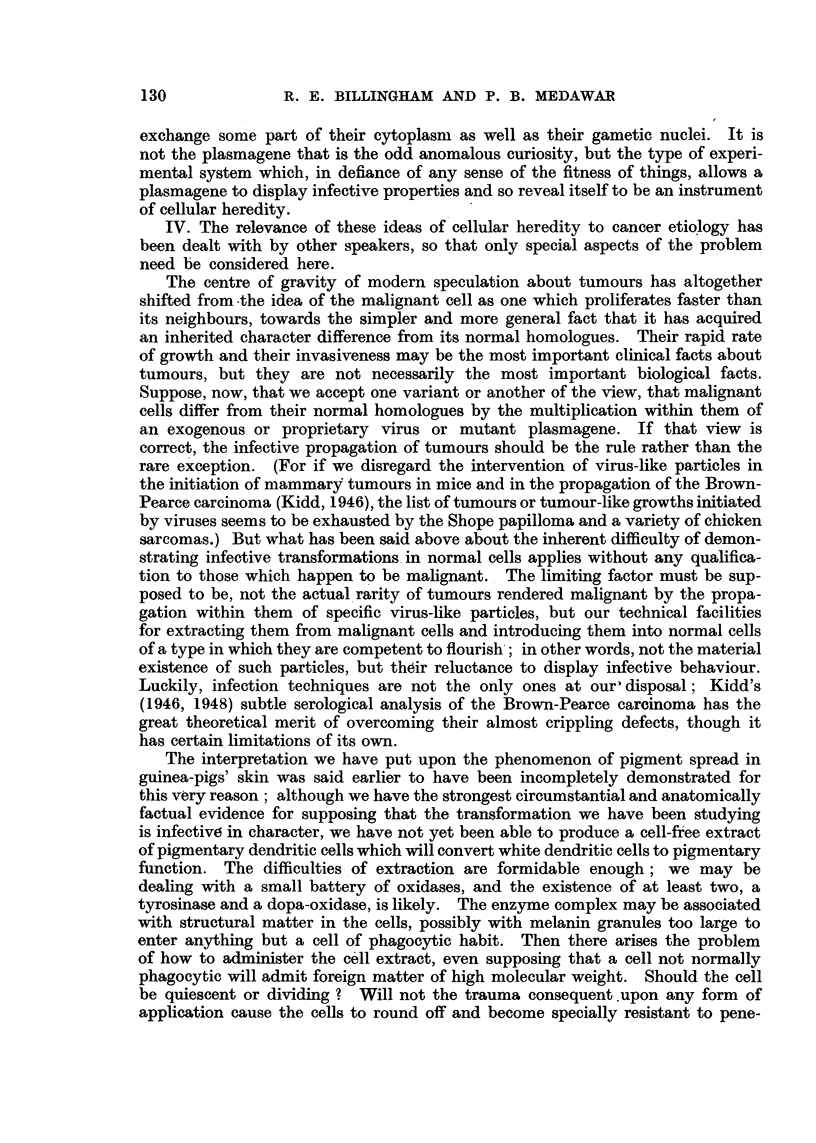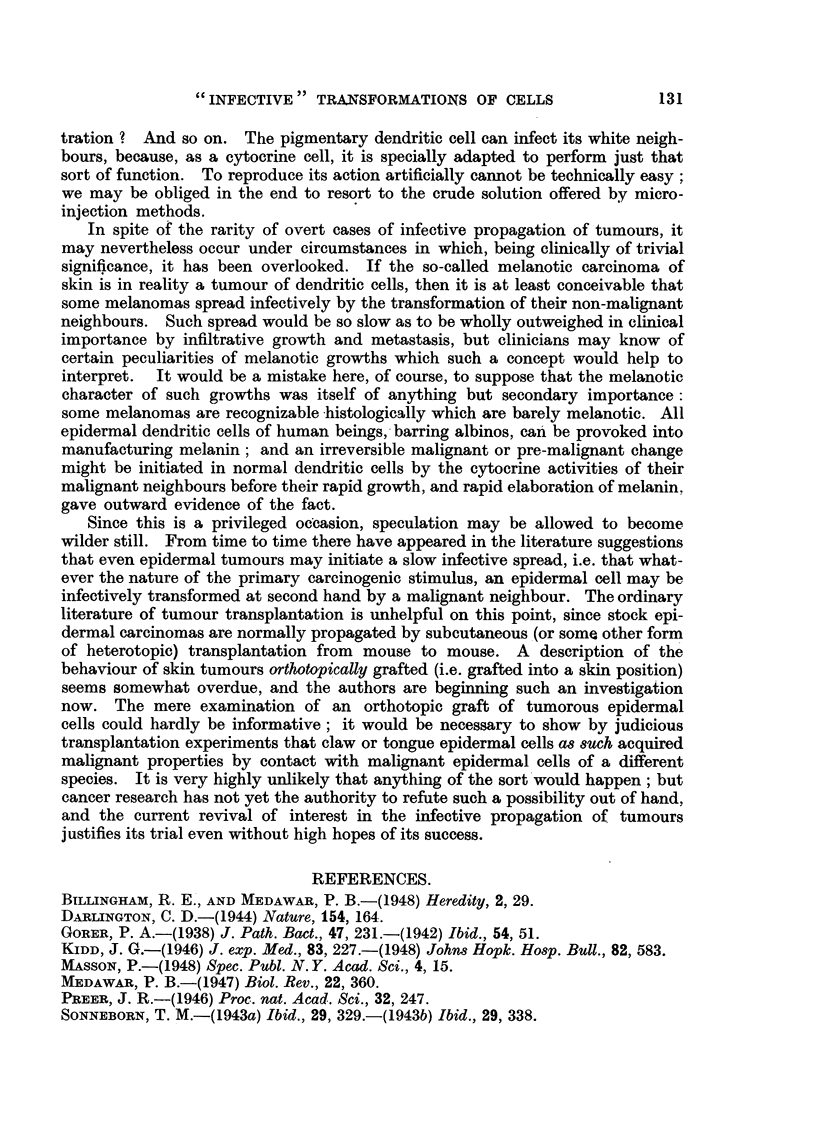# “Infective” Transformations of Cells

**DOI:** 10.1038/bjc.1948.18

**Published:** 1948-06

**Authors:** R. E. Billingham, P. B. Medawar


### " INFECTIVE " TRANSFORMATIONS OF CELLS.

R. E. BILLINGHAM AND P. B. MEDAWAR.
*From the Zoology Department, University of Birmingham.*

Given at the Symposium on the Genetics of Cancer, London, June 24 and 25, 1948.

I. ALTHOUGH biologists have for long been uneasily aware of the existence of a problem of cell heredity, its systematic analysis is the work of very recent years. Cell heredity deals with the origin and maintenance of *inherited* character differences between cells ; more particularly, between cells that set up division lineages by mitotic fission. Its problems present themselves in their most acute, if not most easily workable, form in metazoan development.

The outcome of cellular differentiation in (say) mammalian development is the formation of a limited number of histologically definable cell genera, each one further subdivided into a variety of cellular genetic species. The genus " fibroblast," as yet far from completely analysed, may be subdivided into cells which manufacture bone, cartilage, white connective-tissue fibres, and so on. The genus " epidermis," of which we have made a particular study, includes the epidermal epithelium of the superficial skin, of the sole of the foot, the tongue, the claws or nails, the vagina and the cornea. Cells of each of these epidermal species display a distinctive combination of structural or physiological properties. The epidermis of the sole of the foot, for example, has a characteristically high rate of cell division. One is at first tempted to believe that this is an immediate consequence of the wear and tear and constant irritation that sole epithelium submits to. But this has proved not to be the case. Although plausible " phenocopies " of sole epithelium (in the form of corns and callosities) can be made by irritating the thin and relatively quiescent skin of the body surface, the difference between the division rates of sole and body epidermis is in fact " intrinsic " and inheritable. We have, for example, transplanted sole epidermis to positions in the body where it is protected by neighbouring hair and secure from mechanical irritation ; but even so, its characteristic growth rate has been maintained for at least two years, and thick pads of now functionless cuticle continue to form over it and may periodically be removed. Claw epithelium tells the same story—more clearly, since the difference between claw and body epidermis is anatomically crude and obvious. We have recently begun a study of corneal epithelium, as something of a test case. Corneal epithelium is very transparent, and its mode of cell-packing and cuticle formation is characteristic. These properties might well be the outcome of the peculiar situation in which it lives—lying taut over the unvascularized corneal dermis, relatively low in temperature, and nourished under conditions of low $O_2$ tension by the aqueous humour, and perhaps to some extent through its outer (cuticular) surface as well. If the difference between corneal and body epidermis were merely a difference of "nurture," then the heterotopic transplantation of the corneal epidermis to (say) the chest wall should bring about its reversion to the cell type characteristic of body skin. This does not happen. By grafting small corneal shavings to large granulating areas on the chests of rabbits, the corneal epidermis can be made to expand its substance and area fifty-fold by cell division and outward spread ; but it still retains its characteristic cell packing, mode of cuticle formation and exquisite transparency, so that fine regenerating dermal blood-vessels are to be seen through it as clearly as through a window. Corneal grafts have proved to form a rather unstable epithelium, which never seems to achieve the firm union with the underlying corium characteristic of body skin. For this reason we have not yet been able to "cultivate" corneal epithelium as a heterotopic graft long enough to be quite sure that it represents a distinct epidermal species. We can only say that it has given no hint of reversion after five weeks of cultivation.

In general, the technique of tissue culture confirms the results of heterotopic transplantation ; but it is far less critical in its discrimination between cells of different species, because cultivation *in vitro* has a tendency to make different cells look rather alike. Cultivation *in vitro* is not known to bring about any inherited change in differentiated cells, unless we take into account the fact that prolonged and rapid growth may perhaps cause individual cells to lose specific properties in the "growth rate dilution process" recently reported on by Preer (1946). The "antigenic simplification" (Gorer, 1938, 1942) and anaplastic tendencies of long established and rapidly growing transplanted tumours may conceivably represent a phenomenon of similar origin.

It will be clear from the foregoing examples that the techniques we have so far used to discriminate between the several true-breeding species of epidermal cells are, in their use of heterotopic transplantation, formally identical with those used in classical experimental embryology to distinguish between cell systems undergoing "self" and "dependent" differentiation. The epidermal species illustrate the nature of cell heredity with particular clarity because, unlike the cell systems the embryologist is accustomed to work with, they represent *final terms* of the sequence of embryonic differentiation. The nature of the comparatively small differences between these end-products of differentiation may prove easier to analyse than the radical dichotomies of early embryonic life.

II. The mere contemplation of conservative cell heredity—of a faithfully true-breeding cell lineage—can obviously tell us nothing about the mechanism of cellular inheritance. It is a truism to say that Mendelian genetics is a theory about the *differences* between individuals. Mendelian genetics (from which we exclude comparative cytogenetics) has nothing to say about any character common to all the members of an interbreeding assembly ; there is a genetical theory about the ability to taste phenyl thio-urea, but not about the ability to taste salt. (It is most unfortunate that the phylum- and class-specific characters of animals belong to the group on which Mendelian theory is, for technical reasons, obliged to remain silent ; from this, no doubt, arises the lay misconception that Mendelism only " accounts for " relative trivialities.) In short, Mendelian theory is made possible by the phenomenon of segregation. The study of cell heredity is likewise, in effect, the study of cellular transformation—that is, of the origin of inherited differences between the phenotypes of cells.

From our point of view, the most important of the several different types of cell transformation is that which, in a rough preliminary classification, has been called *infective* (Medawar, 1947). To this category belong the transformations of cells by endogenous or exogenous viruses or virus-like bodies. Infective transformations have two distinctive features. The first is that the maintenance of the transformed state they bring about depends upon the continued presence in the cell of the transforming agent or a faithful copy of it. The second, a corollary of the first, is that infective transformations can be serially propagated —a cell once transformed can in its turn transform another.

The special importance of infective transformations is that they provide clear direct evidence of the *particulate* nature of cell heredity, and so, indirectly, of the fact that the differences between cell species are combinatorial in nature and not smoothly blended. The authors' work on the inheritance of colour differences in the cells of guinea-pigs' skin is relevant here. In spotted guinea-pigs (as, apparently, in spotted pigs and Friesian cattle) the pigmentation of the black skin areas can be seen during life to encroach upon the white. The spread is even in density and smoothly progressive, but it normally leaves untouched the hairs of the white area that are being encroached upon, so that the transitional zone of black skin that was formerly white is distinguished by bearing white hairs on a black skin background. Our interpretation of the phenomenon (Billingham and Medawar, 1948) is, in outline, as follows: The origin and seat of pigmentary function in black or other coloured skins is the *pigmentary dendritic cell*, a branching cell which lives in the lower reaches of the epidermis and which in some unexplained manner " feeds "—or, as Masson (1948) puts it, " injects " —melanin granules into the Malpighian layer cells on which its branches end. Pigmentary dendritic cells form a facultatively syncytial system, since the branches of neighbouring cells are sometimes confluent, and a branch from one dendritic cell, instead of ending on a Malpighian cell, may sometimes apply itself to the cell body of another.

In the white areas a dendritic cell system is present which is similar in every respect save one : the cells lack both melanin granules and the enzyme apparatus required for making it, and no form of merely physical stimulus will cause them to acquire pigmentary function.

We have shown, not yet with final precision, that the phenomenon of pigment encroachment or spread at the pigmentation boundaries of spotted guinea-pigs is due to the transformation of white (non-pigmentary) dendritic cells into melanin-forming cells by contact with their pigmentary neighbours. The transformation, once achieved, is permanent in the transformed cell and its division lineage ; and a white cell transformed to pigmentary function can in its turn transform its neighbours. The process of pigment spread is thus contagiously infective ; and as a crude first approximation we have suggested that the infective agent is a " plasmagene " (Darlington, 1944) type of enzyme or enzyme-complex with (in this context) a virus-like behaviour. According to our interpretation, therefore, the inherited difference between pigmentary and non-pigmentary dendritic cells proves to satisfy the following very simple equation :

pigmentary dendritic cell = non-pigmentary dendritic cell + pigment-forming system.

III. We have now to ask whether the pigment-spread phenomenon we have analysed is a mere curiosity of nature, or whether it exemplifies a principle of general importance.

The experimental system we have been using is a very unusual one indeed, for the following reasons : Most cells liberate their secretory products externally or into the general circulation. Dendritic cells pass *their* secretory product, melanin, into the cells around them—" cytocrine " activity, as Masson calls it. Their neighbours are normally the Malpighian cells of the epidermis ; but, as Masson has pointed out, dendritic cells may also inject melanin granules into cells of quite different origin—into the cells of gut epithelium for example, if a tumour metastasis happens to bring them within reach of its branches. In all such cases the melanin is acquired passively or at second hand, and behaves as an inert end-product. Pigment spread becomes possible only when pigmentary dendritic cells are given access to non-pigmentary dendritic cells, and these are so little different from their pigmentary neighbours that they are " competent," as embryologists say, to support the continued formation of the pigment-producing enzyme complex presumably introduced into them. Since skin homografts do not survive, so that black skin from one individual cannot be made without any special techniques to initiate pigment spread in the white skin of another, the phenomenon can only be studied in spotted animals. But not in all laboratory animals—only in those, like the guinea-pig, and unlike the rabbit or mouse, in which dendritic cells are present in the superficial epidermis. (Pigment spread does not occur in spotted rabbits or mice.) In short, an infective transformation of the type we are considering is made possible only by the coexistence in one individual of a semi-syncytial system of cytocrine cells of two species, differing from one another in a single, specific, and at the same time phenotypically conspicuous way. On the face of it, nothing could be more " specialized " or more trivially parochial in its significance.

Further reflection shows that such an interpretation would be short-sighted. It may well be that the cytoplasmic enzymes which endow the various cell species with their characteristic " phenotypic " properties are particulate self-reproducing entities ; but this cannot be *demonstrated* unless such an entity is removed from one cell and transplanted into and caused to multiply within some other cell competent to give it quarter. But the whole mechanism of development can be regarded as a conspiracy to prevent just this sort of thing's happening. The casual leakage of enzymes from one cell into another would make nonsense of embryological development ; no organ could exist under these conditions, with its great variety of cells of different types in intimate physical contact. An infective transformation can only be secured by taking advantage of peculiar cell properties, like the cytocrine properties of dendritic cells and their transiently syncytial lay-out ; of peculiar animals, like spotted guinea-pigs ; or of technical tricks, like grafting claw epithelium to the skin of the chest, or—to choose an example from the illuminating pioneer work of Sonneborn (1943*a*, *b*)—by delaying the conjugation process of *Paramecium* so that the conjugant pairs may exchange some part of their cytoplasm as well as their gametic nuclei. It is not the plasmagene that is the odd anomalous curiosity, but the type of experimental system which, in defiance of any sense of the fitness of things, allows a plasmagene to display infective properties and so reveal itself to be an instrument of cellular heredity.

IV. The relevance of these ideas of cellular heredity to cancer etiology has been dealt with by other speakers, so that only special aspects of the problem need be considered here.

The centre of gravity of modern speculation about tumours has altogether shifted from the idea of the malignant cell as one which proliferates faster than its neighbours, towards the simpler and more general fact that it has acquired an inherited character difference from its normal homologues. Their rapid rate of growth and their invasiveness may be the most important clinical facts about tumours, but they are not necessarily the most important biological facts. Suppose, now, that we accept one variant or another of the view, that malignant cells differ from their normal homologues by the multiplication within them of an exogenous or proprietary virus or mutant plasmagene. If that view is correct, the infective propagation of tumours should be the rule rather than the rare exception. (For if we disregard the intervention of virus-like particles in the initiation of mammary tumours in mice and in the propagation of the Brown-Pearce carcinoma (Kidd, 1946), the list of tumours or tumour-like growths initiated by viruses seems to be exhausted by the Shope papilloma and a variety of chicken sarcomas.) But what has been said above about the inherent difficulty of demonstrating infective transformations in normal cells applies without any qualification to those which happen to be malignant. The limiting factor must be supposed to be, not the actual rarity of tumours rendered malignant by the propagation within them of specific virus-like particles, but our technical facilities for extracting them from malignant cells and introducing them into normal cells of a type in which they are competent to flourish; in other words, not the material existence of such particles, but their reluctance to display infective behaviour. Luckily, infection techniques are not the only ones at our disposal; Kidd's (1946, 1948) subtle serological analysis of the Brown-Pearce carcinoma has the great theoretical merit of overcoming their almost crippling defects, though it has certain limitations of its own.

The interpretation we have put upon the phenomenon of pigment spread in guinea-pigs' skin was said earlier to have been incompletely demonstrated for this very reason; although we have the strongest circumstantial and anatomically factual evidence for supposing that the transformation we have been studying is infective in character, we have not yet been able to produce a cell-free extract of pigmentary dendritic cells which will convert white dendritic cells to pigmentary function. The difficulties of extraction are formidable enough; we may be dealing with a small battery of oxidases, and the existence of at least two, a tyrosinase and a dopa-oxidase, is likely. The enzyme complex may be associated with structural matter in the cells, possibly with melanin granules too large to enter anything but a cell of phagocytic habit. Then there arises the problem of how to administer the cell extract, even supposing that a cell not normally phagocytic will admit foreign matter of high molecular weight. Should the cell be quiescent or dividing? Will not the trauma consequent upon any form of application cause the cells to round off and become specially resistant to penetration ? And so on. The pigmentary dendritic cell can infect its white neighbours, because, as a cytocrine cell, it is specially adapted to perform just that sort of function. To reproduce its action artificially cannot be technically easy ; we may be obliged in the end to resort to the crude solution offered by microinjection methods.

In spite of the rarity of overt cases of infective propagation of tumours, it may nevertheless occur under circumstances in which, being clinically of trivial significance, it has been overlooked. If the so-called melanotic carcinoma of skin is in reality a tumour of dendritic cells, then it is at least conceivable that some melanomas spread infectively by the transformation of their non-malignant neighbours. Such spread would be so slow as to be wholly outweighed in clinical importance by infiltrative growth and metastasis, but clinicians may know of certain peculiarities of melanotic growths which such a concept would help to interpret. It would be a mistake here, of course, to suppose that the melanotic character of such growths was itself of anything but secondary importance : some melanomas are recognizable histologically which are barely melanotic. All epidermal dendritic cells of human beings, barring albinos, can be provoked into manufacturing melanin ; and an irreversible malignant or pre-malignant change might be initiated in normal dendritic cells by the cytocrine activities of their malignant neighbours before their rapid growth, and rapid elaboration of melanin, gave outward evidence of the fact.

Since this is a privileged occasion, speculation may be allowed to become wilder still. From time to time there have appeared in the literature suggestions that even epidermal tumours may initiate a slow infective spread, i.e. that whatever the nature of the primary carcinogenic stimulus, an epidermal cell may be infectively transformed at second hand by a malignant neighbour. The ordinary literature of tumour transplantation is unhelpful on this point, since stock epidermal carcinomas are normally propagated by subcutaneous (or some other form of heterotopic) transplantation from mouse to mouse. A description of the behaviour of skin tumours *orthotopically* grafted (i.e. grafted into a skin position) seems somewhat overdue, and the authors are beginning such an investigation now. The mere examination of an orthotopic graft of tumorous epidermal cells could hardly be informative ; it would be necessary to show by judicious transplantation experiments that claw or tongue epidermal cells *as such* acquired malignant properties by contact with malignant epidermal cells of a different species. It is very highly unlikely that anything of the sort would happen ; but cancer research has not yet the authority to refute such a possibility out of hand, and the current revival of interest in the infective propagation of tumours justifies its trial even without high hopes of its success.

### REFERENCES.

Billingham, R. E., and Medawar, P. B.—(1948) *Heredity*, **2**, 29.
Darlington, C. D.—(1944) *Nature*, **154**, 164.
Gorer, P. A.—(1938) *J. Path. Bact.*, **47**, 231.—(1942) *Ibid.*, **54**, 51.
Kidd, J. G.—(1946) *J. exp. Med.*, **83**, 227.—(1948) *Johns Hopk. Hosp. Bull.*, **82**, 583.
Masson, P.—(1948) *Spec. Publ. N.Y. Acad. Sci.*, **4**, 15.
Medawar, P. B.—(1947) *Biol. Rev.*, **22**, 360.
Preer, J. R.—(1946) *Proc. nat. Acad. Sci.*, **32**, 247.
Sonneborn, T. M.—(1943*a*) *Ibid.*, **29**, 329.—(1943*b*) *Ibid.*, **29**, 338.